# Supplementary material for: The Impact of Gold Nanoparticles on Somatic Embryogenesis Using the Example of Arabidopsis thaliana
Source: Int J Mol Sci. 2023 Jun 19;24(12):10356. doi: 10.3390/ijms241210356 (PMC10299670; doi:10.3390/ijms241210356)
Supplement: Supplementary file 1 [file ijms-24-10356-s001.zip › ijms-2425463-supplementary.pdf]

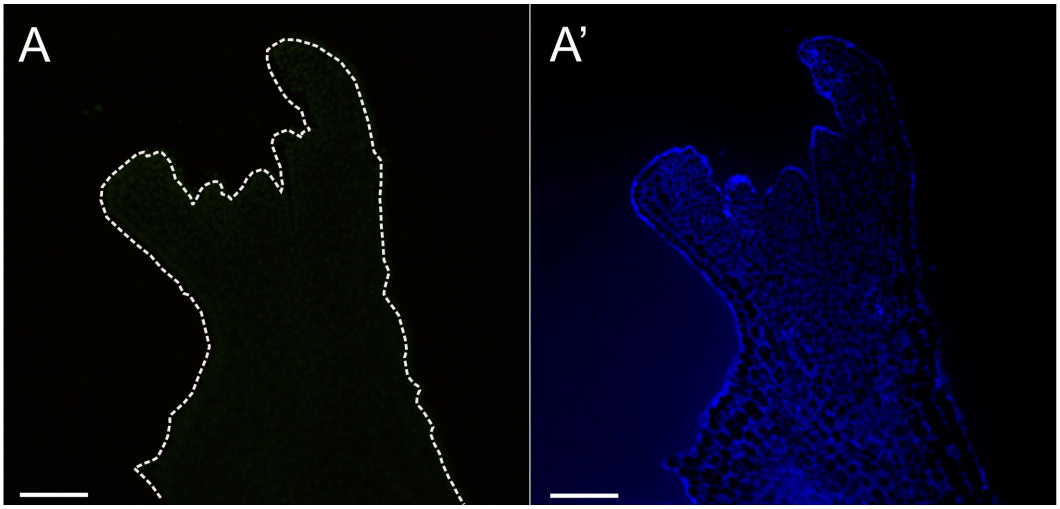

Figure S1. A- negative control of BPEI Au NPs-treated explant and the same section from A staining with calcofluor. Scale bars—200 μm.
